# Pattern Electroretinography Changes in Patients with Established or Suspected Primary Open Angle Glaucoma

**DOI:** 10.5005/jp-journals-10008-1135

**Published:** 2013-05-09

**Authors:** Sunil Ganekal, Syril Dorairaj, Vishal Jhanji

**Affiliations:** Nayana Super Speciality Eye Hospital and Research Center Davangere, Karnataka, India; Department of Ophthalmology, Mayo Clinic Jacksonville, Florida USA; Department of Ophthalmology and Visual Sciences, Chinese University of Hong Kong, Hong Kong

**Keywords:** Pattern ERG, Primary open angle glaucoma, Glaucoma suspects.

## Abstract

**Purpose:** To assess pattern electroretinogram (PERG) changes in patients with established or suspected primary open angle glaucoma (POAG).

**Materials and methods:** Transient PERG using LV Prasad eye electrodes were performed in 76 normal, 32 glaucomatous and 22 glaucoma suspect eyes. The P50 amplitude, N95 amplitude and P50 latency were analyzed. The results were further analyzed with receiver operating characteristic (ROC) curves and discriminant function analysis (DFA).

**Results:** The P50 and N95 amplitude of the POAG and glaucoma suspect groups were significantly reduced. There was significant shortening in the P50 latency in the POAG and glaucoma suspect groups. DFA using the P50 amplitude, N95 amplitude and P50 latency waveform parameters showed a sensitivity and specificity of 76.67 and 88.57% respectively.

**Conclusion:** Pattern ERG demonstrated significant changes in POAG patients and suspects. ROC curves for the three wave parameters demonstrated that N95 amplitude was the better indicator for diagnosis of POAG when used individually.

**How to cite this article:** Ganekal S, Dorairaj S, Jhanji V. Pattern Electroretinography Changes in Patients with Established or Suspected Primary Open Angle Glaucoma. J Current Glau Prac 2013;7(2):39-42.

## INTRODUCTION

The pattern electroretinogram (PERG) is a retinal biopotential evoked by a temporally modulated patterned stimulus (e.g. checkerboard or grating) of constant mean luminance. Pattern ERG allows both a measure of central retinal function, and, in relation to its origins, an evaluation of retinal ganglion cell function and also an evaluation of macular dysfunction.^1^

There are evidences to suggest that large diameter ganglion cells which produce PERG are affected earlier than small ganglion cells in the evolution of glaucoma.^2,3^ PERG changes can help detecting early glaucomatous damage. PERG recorded from glaucomatous eyes that had no field defect within the retinal area covered by the PERG stimulus, a pathological PERG were recorded in approximately 71% of such eyes.^4^ Serial PERG's can have a place in management of primary open angle glaucoma (POAG) patients and glaucoma suspects.^5^

Previous literature suggests that PERG changes occur in glaucoma suspects who at a later date progress to have POAG.^5^ PERG recordings are done with contact electrodes that contact the cornea or bulbar conjunctiva. A cheap but effective and safe conjunctival contact fiber electrode derived from Zeri (LV Prasad eye electrode) was used in our study. PERG recording with the LVP eye electrode in normal subjects is reported.^6^ Not much literature is available in India analyzing PERG changes in POAG. The changes in PERG, in POAG and suspects were prospectively analyzed using LVP eye electrodes.

## MATERIALS AND METHODS

All patients who were diagnosed to have POAG (patients with at least two of the following criteria: visual field defect not explainable by other causes, CDR ≥ 0.8 or an asymmetry in CDR ≥ 0.2, IOP ≥ 22 mm Hg, or IOP lowering treatment) (22 eyes of 11 patients), glaucoma suspects (one of the following in at least one eye in an individual with open anterior chamber angles by gonioscopy; appearance of the optic disk or retinal nerve fiber layer that is suspicious for glaucomatous damage, a visual field suspicious for glaucomatous damage in the absence of clinical signs of other optic neuropathies or consistently elevated IOP associated with normal appearance of the optic disk and retinal nerve fiber layer and with normal visual field test results) (32 eyes of 16 patients) and normal volunteers (76 eyes of 38 patients) were selected from patients attending the outpatient Department of Nayana Super Specialty Eye Hospital and Research Center between July 2011 and December 2011 were randomly enrolled in the study. The study was approved by the institutional review board. Informed consent was taken from all the individuals who participated in the study. Patients with refractive errors ≥ 9D, advanced cataract, uveitis opaque hazy media, diabetic retinopathy, optic nerve head disease of any other etiology, retinal pigment layer disorders and rod or cone dystrophies were excluded from the study. All patients underwent detailed ophthalmic evaluation which included measurement of best corrected visual acuity, intraocular pressure measurement, gonioscopy, fundus evaluation by indirect ophthalmoscopy and slit-lamp biomicroscopy, visual field analysis was done in POAG and glaucoma suspects by Humphrey 30-2 test. Transient PERG recording was done in all the subjects. Transient pattern ERG was recorded using recording electrode (LVP), reference electrode (silver chloride) and ground electrode (silver chloride) in accordance with ISCEV (International Society for Clinical Electrophysiology of Vision) guidelines.

Patient was seated comfortably on a chair at a distance of 1 meter. Pupils were not dilated and subjects were given appropriate spectacle correction for near vision. Black and white reversing checkerboard stimulus was given with a 12-inch television monitor. It covered a field size of 21.6° and the check size was 2.7°. Contrast between black and white squares was greater than 95%. Photopic luminance level of white areas was 80 candela/square meter and overall screen luminance did not vary during checkerboard reversals. Frame rate maintained with raster based CRT of frequency of 75 Hz. Background dim room illumination was used for the entire recordings. A reversal rate of 6 reversals/second (3 Hz) with a sweep time of 150 ms was used. Computerized artifact rejection was set at 100 μV. Monocular recording was done. 150 responses were averaged and two or three full recordings of each stimulus condition were obtained. The recording with the least noise was selected, marked and printouts were obtained.

## RESULTS

**Fig. 1:**
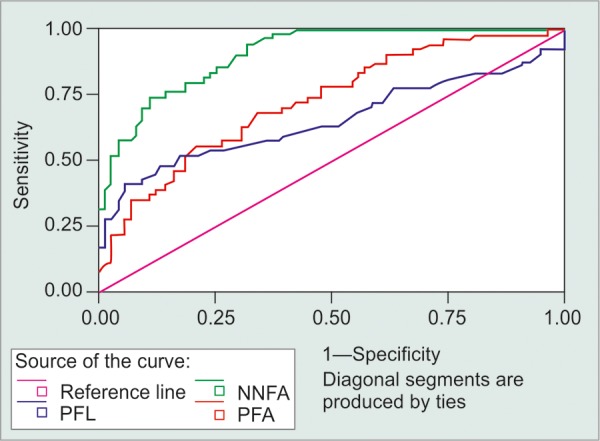
ROC curve of three waveform (PFA: P50 amplitude, NNFA: N95 amplitude, PFL: P50 latency)

Mean age of the individuals participated in the study was 47.83 ± 10.20 years (range: 28-65 years). There were 34 males and 31 females in the study. There was a significant difference in the mean P50 amplitude and N95 amplitude of the three groups (p = 0.0013, p = 0.0012). The mean P50 latency was statistically significant (p = 0.0012). No significant difference was seen between the mean P50 amplitudes, N95 amplitude and P50 latency of the POAG and glaucoma suspect groups (p = 0.974, p = 0.190 and p = 0.974 respectively). Significant difference was observed between the P50 and N95 amplitudes of the normal and raised intraocular pressure group (p = 0.013). The mean P50 latency was significantly lesser in subjects with raised IOP (p = 0.02). Receiver operating characteristic (ROC) curves was used to analyze the sensitivity and specificity of the three wave (P50 amplitude, N95 amplitude and P50 latency) parameters. There was a significant difference in the areas under the three curves, with an overall p-value = 0.00005 and significant individual differences between areas under N95 amplitude *vs* P50 amplitude (p = 0.0009) and areas under N95 amplitude *vs* P50 latency (p = 0.000009) was also observed. But there was no significance in the areas under the ROC curves for P50 amplitude and P50 latency, p-value = 0.22 (Fig. 1). Therefore, N95 amplitude can be considered a better parameter for diagnosis of glaucoma in comparison to P50 amplitude and P50 latency. In order to obtain a comprehensive equation for classification of an individual into normal and abnormal (POAG and glaucoma suspects) categories based on the three wave parameters alone, the discriminant function analysis (DFA) was used. It was observed that 81.6% cases of normals were identified as normals and 85.2% cases of abnormal (POAG and glaucoma suspects) were identified as abnormals. Using the DFA, a sensitivity of 76.67% and specificity of 88.57% was observed.

## DISCUSSION

PERG changes in POAG patients and suspects have been documented.^5,7^ Initial reports suggested that the entire PERG waveform is affected by ganglion cell destruction.^8,9^ But the concepts changed with time. PERG recording in normal subjects is reported to be ranging from 0.5 to 8 μV.^10^ Since, it is a small voltage response, recordings can be contaminated by noise and artifacts.^5^ Hence, in this study, two to three recordings were taken per eye to select the waveform with minimal contamination.

PERG to large stimulus checks is reported to be relatively normal in early glaucoma whereas PERG to smaller check sizes are affected in early and late glaucoma.^11^ In our study small check sizes were used. All subjects were corrected to the testing distance as delayed latency and decreased waveform amplitude has been reported with 2D of refractive error.^7^

There was a statistically significant reduction in the P50 amplitude in both the glaucoma suspect and the POAG groups when compared with normals. Results of this study are consistent with that of E O'Donaghue et al^5^ and Neoh et al^12^ who also noted a reduction in P50 amplitude in the POAG patients.^5^

A significant reduction in the N95 amplitudes was noted in our glaucoma suspects and POAG patients. Peter Wanger et al^7^ reported that the amplitude of N95 was reduced more in the glaucomatous eyes in patients with unilateral glaucoma. E O'Donaghue et al^5^ and Neoh et al^12^ reported after serial PERG's that N95 amplitude assessment allows separation of patients with glaucoma, ocular hypertension and no disease. Ruben et al^13^ and Michael Bach et al^11^ also reported that there is reduction in N95 amplitude of transient PERG in early glaucoma patients. Hence, results of this study are comparable to other human and animal studies in literature.

In our study, there was statistically significant reduction of P50 amplitude, N95 amplitude and P50 latency in abnormal group. E O'Donaghue et al reported that PERG recordings varied between normals, glaucoma suspects and POAG patients.^5^ E O'Donaghue et al showed that PERG amplitudes of both P50 and N95 reduced progressively from the normal group to the raised IOP group.^5^ Michael Bach reported that IOP is a major risk factor for developing glaucoma.^11^ Hence, the results of this study that PERG waveform decreases with increase in IOP are comparable. In our study, among the above three parameters analyzed by ROC, N95 amplitude was found to be most sensitive parameter. Graham et al^14^ reported that in PERG, N95 amplitude was the most sensitive parameter for evaluation of glaucoma. There was a significant difference between the areas under N95 amplitude and P50 amplitude as well as N95 amplitude and P50 latency.

Out of 76 eyes classified normal by clinical assessment, 62 eyes (81.6%) were identified as normal by using the cutoff value of the discriminant analysis. Similarly, out of 54 eyes classified clinically as glaucoma suspects and POAG patients, 46 eyes (85.2%) were identified as abnormal by using the cutoff value. The sensitivity and specificity was calculated to be 76.67 and 88.57% respectively. Pfeiffer et al reported a sensitivity of 82.7% and a specificity of 90.8% using discriminant analysis.^15^ Hence, the results of our study are comparable with results of other studies in literature.

Limitations of the present study were; transient PERG recording in our study was done monocularly. Binocular recording has the advantages that it is more stable, reduces examination time and allows better fixation in cases of asymmetric visual loss. In addition, if simultaneous recording is done, the background parameters will be exactly the same. Although transient PERG demonstrates changes in POAG patients and suspects, steady state PERG is reported to demonstrate more changes. The bigger sample size would have been ideal and the results obtained then could have been more conclusive. Although all the three wave parameters *viz* P50 amplitude, N95 amplitude and P50 latency were significantly affected in the glaucoma suspect group, to be conclusive that some or most of these patients will at a later date develop POAG, demonstration of visual field defect on long-term follow-up would have been ideal.

## CONCLUSION

PERG recording is a useful objective test to analyze the functioning of the ganglion cell layer of the retina. PERG picks up a pan retinal damage of the ganglion cells earlier to the occurrence of visual field defects.
